# Decoding *SCN2A* Variants: Bridging Genetics and Phenotypes in Autism Spectrum Disorder

**DOI:** 10.3390/jcm14113790

**Published:** 2025-05-28

**Authors:** Nicholas DiStefano, Jaimee N. Cooper, David H. Elisha, Max Zalta, Jeenu Mittal, David Cohen, Andrea Monterrubio, Ryan Hossain, Akhila Sangadi, Rahul Mittal, Adrien A. Eshraghi

**Affiliations:** 1 Hearing Research and Communications Disorders Laboratory, Department of Otolaryngology, University of Miami Miller School of Medicine, 1600 NW 10th Avenue, Miami, FL 33136, USA; nvd10@miami.edu (N.D.); jcooper12@student.nymc.edu (J.N.C.); 77mrzalta@gmail.com (M.Z.); j.mittal@med.miami.edu (J.M.); dhc65@med.miami.edu (D.C.); andream.monterrubio@gmail.com (A.M.); rhoss014@med.fiu.edu (R.H.); asangadi@med.miami.edu (A.S.); r.mittal11@med.miami.edu (R.M.); 2 School of Medicine, New York Medical College, Valhalla, NY 10595, USA; 3 Faculty of Medicine, Tel Aviv University, Tel Aviv 6997801, Israel; 4 Herbert Wertheim College of Medicine, Florida International University, Miami, FL 33199, USA; 5 Department of Neurological Surgery, University of Miami Miller School of Medicine, 1600 NW 10th Avenue, Miami, FL 33136, USA; 6 Department of Biomedical Engineering, University of Miami, Coral Gables, FL 33146, USA; 7 Department of Pediatrics, University of Miami Miller School of Medicine, 1600 NW 10th Avenue, Miami, FL 33136, USA

**Keywords:** autism spectrum disorder, *SCN2A*, Nav1.2 sodium channel, neurodevelopmental disorders, genotype–phenotype correlation, mosaicism, targeted therapy, genetic counseling

## Abstract

**Background:** Autism spectrum disorder (ASD) is a complex neurodevelopmental condition with a rising prevalence, driven by multifactorial genetic and environmental factors. Among the genetic contributors identified, *SCN2A*, a critical gene encoding the Nav1.2 sodium channel, has been implicated in ASD and other related neurological conditions. This systematic review aims to explore the relationship between *SCN2A* mutations and ASD phenotypes. **Methods:** This review systematically analyzed data from studies reporting *SCN2A* mutations in individuals diagnosed with ASD. The primary focus was on the characterization of mutation types, associated clinical features, and phenotypic variability. **Results:** The mutations identified were predominantly *de novo* missense mutations and were associated with a spectrum of neurological and developmental challenges, including seizures, intellectual disability, movement disorders, and repetitive behaviors. A notable finding was the significant phenotypic variability observed across individuals. Gender differences emerged, suggesting a potentially greater impact on females compared to trends typically seen in ASD genetic studies. Specific mutations, such as c.2919+4delT, and mosaicism were identified as novel contributors to the observed heterogeneity. **Conclusions:** The review highlights the clinical significance of *SCN2A* mutations in ASD and highlights their relevance in genetic counseling and the development of targeted therapies. Understanding the diverse genotype–phenotype correlations associated with *SCN2A* can drive progress in personalized medicine, paving the way for precision therapies tailored to individuals with *SCN2A*-related ASD.

## 1. Introduction

Autism spectrum disorder (ASD) is a neurodevelopmental condition defined by a constellation of social communication difficulties and restricted, repetitive behaviors. According to the most recent edition of the Diagnostic and Statistical Manual of Mental Disorders (DSM-V-TR) [1], ASD is defined by persistent deficits in social communication and interaction across multiple contexts; restricted and repetitive patterns of behavior, interests, or activities; symptoms that are present in the early developmental period; clinically significant impairments in social, occupational, or other areas of functioning; and symptoms that are not better explained by intellectual disability or global developmental delay. These criteria also acknowledge the heterogeneity in the clinical presentation of ASD, recognizing that it may occur with or without accompanying intellectual or language impairments, and it can vary in severity based on the level of support required. Importantly, while many individuals with ASD exhibit developmental delays, not all phenotypes follow this trajectory. Recent evidence indicates that some children may not show early developmental concerns but instead exhibit social communication differences or behavioral traits that emerge over time [2].

ASD is a highly heterogeneous condition, with symptoms ranging from mild to severe, affecting individuals differently across the spectrum [3,4]. Additionally, many individuals with ASD exhibit atypical behaviors such as obsessive attachments, hypersensitivity to sensory stimuli, and repetitive actions such as body rocking, hand flapping, and finger flicking [3,5,6,7,8,9]. These behaviors can significantly impact daily functioning and quality of life, underscoring the importance of early diagnosis and intervention [10,11,12].

Early identification is critical for improving outcomes in individuals with ASD. Research shows that those diagnosed at a younger age are more likely to develop higher IQ scores, achieve greater levels of independence, and exhibit better adaptive skills later in life [5,13,14,15,16]. Current interventions include a combination of physical and occupational therapies, alongside tailored medical and behavioral treatments, aimed at addressing the diverse needs of individuals with ASD and their comorbidities [3,17,18,19,20,21,22,23].

Emerging evidence has linked ASD to complex interactions between genetic predispositions and environmental influences, which affect key neurodevelopmental processes such as synaptic signaling, neural connectivity, and brain plasticity. Recent advances in genetic and epigenetic research have begun to uncover the molecular pathways involved in ASD, including disruptions in regulatory networks that shape early brain development [23,24,25,26,27,28,29]. These findings highlight the critical need for a multidisciplinary approach to deepen our understanding of ASD’s underlying mechanisms.

As ASD diagnoses continue to rise, with approximately 1 in 36 children in the United States affected, it is imperative to advance research into its genetic and epigenetic foundations [30,31]. Integrating insights from molecular biology, neuroscience, and clinical studies is essential for developing targeted therapies that address the underlying causes of ASD, ultimately improving outcomes and quality of life for individuals with this complex disorder [30]. The etiology of ASD is multifactorial, with many genes contributing to the pathophysiology and prognosis of this condition [30,32,33]. An open-access database from the Simons Foundation Autism Research Initiative (SFARI) (https://www.sfari.org/resource/sfari-gene, accessed on 16 March 2025) was utilized due to its highly precise and accurate criterion to assess the role of various genes involved in ASD. Gene scoring is used to appraise the strength of correlation between that gene and ASD diagnosis or development risk. A designation of 1–3 is given, where 1 indicates a gene with significant implication in ASD with at least 3 reported likely de novo gene-disrupting mutations. Each of those signifies meeting a threshold false discovery rate of <0.1. A designation of 2 is given to genes with two reported gene-disrupting de novo mutations, and a designation of 3 is given to genes with one. Genes with a designation of syndromic (S) can be assigned if genes carry a considerably high risk for ASD. In order to establish a causative relationship between a gene and ASD diagnosis, an Evaluation of Autism gene Link Evidence (EAGLE) score can be utilized, where increased scores indicate a greater likelihood of causation of ASD https://gene.sfari.org/ (accessed on 4 November 2024. Among the numerous genes identified as being significant for contributing to the development of ASD, *SCN2A* has been identified as a category 1 gene, with the second highest EAGLE score of 109.3.

**Figure 1:**
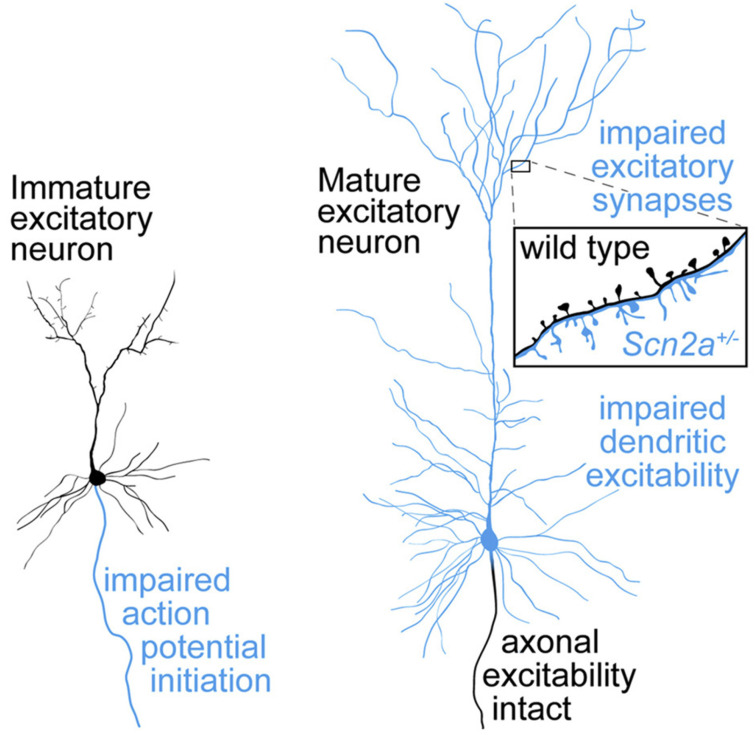
**Schematic illustration of *SCN2A* haploinsufficiency disrupting neuronal excitability across development.** In immature excitatory neurons, loss of NaV1.2 disrupts action potential initiation. In mature neurons, dendritic excitability and excitatory synaptic function are impaired despite intact axonal excitability. Adapted with permission from Spratt et al. [17].

**Figure 2:**
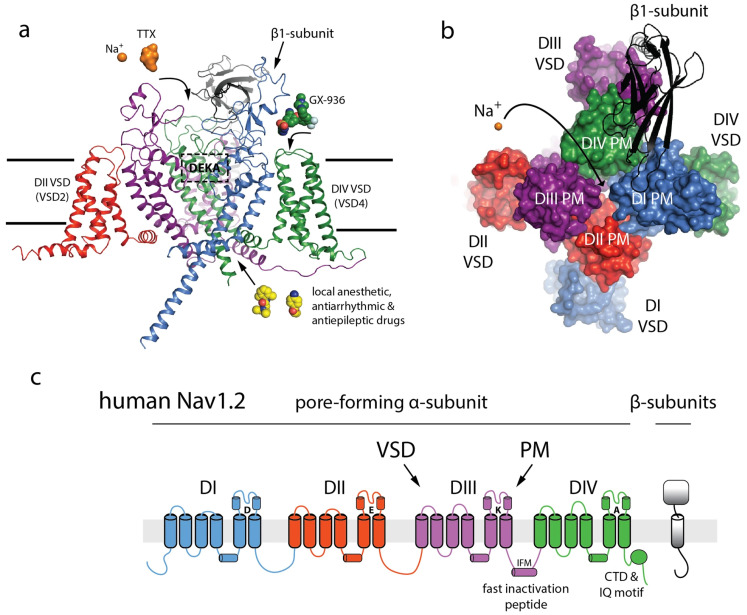
**Structure, architectural elements, and sodium channel (NaV) topology.** (**a**) Side view of the α-subunit with color-coded domains (DI–DIV) and β1-subunit (gray). TTX, Na^+^, and GX-936 binding sites are shown. (**b**) Top view illustrating the central pore and domain-swapped architecture. (**c**) Schematic of NaV1.2 topology, highlighting voltage-sensing domains (VSDs), pore modules (PMs), and regulatory motifs. Taken from Kruth et al. [39] under a Creative Commons Attribution 4.0 International License, which permits use, sharing, adaptation, distribution, and reproduction in any medium or format.

**Figure 3:**
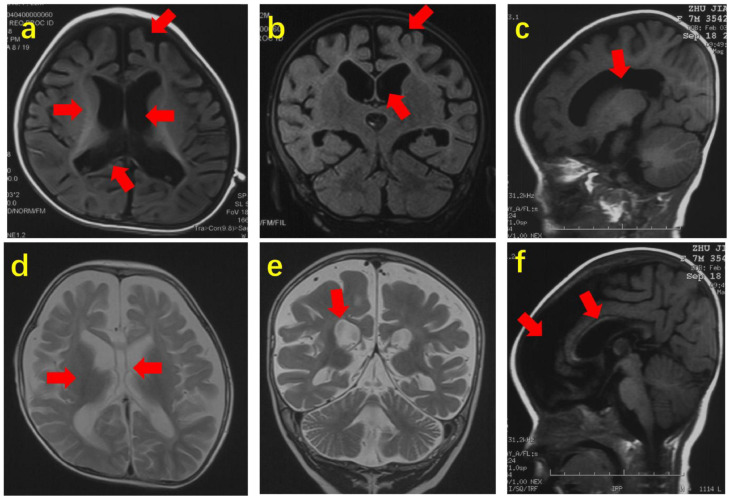
**Brain MRI abnormalities in patients with SCN2A variants.** (**a**,**b**) Axial and coronal T1-weighted images (Patient 14, age 22 months) showing corpus callosum agenesis, delayed myelination, frontotemporal dysplasia, and ventricular enlargement. (**c**,**f**) Sagittal T1-weighted images (Patient 29, age 7 months) showing similar findings. (**d**,**e**) Axial and coronal T2-weighted images (Patient 39, age 12 months) showing corpus callosum agenesis, delayed myelination, and ventricular enlargement. Arrows indicate lesion sites. Taken from Zeng et al. [51] under the terms of the Creative Commons Attribution License (CC BY).

Several studies have identified *SCN2A* as a key genetic contributor to ASD development [3,17,34,35,36,37,38,39,40,41,42]. *SCN2A* mutations in ASD significantly impair the initiation and propagation of neuronal action potentials, as well as axonal excitability, which are critical for proper early neurodevelopment. These disruptions compromise dendritic and synaptic functions, resulting in diminished synaptic plasticity and strength, key processes essential for effective neuronal communication and cognitive function (Figure 1) [43]. In fact, a 2018 study identified that 0.3% of children with ASD have the *SCN2A* variant [44]. The gene is located on the positive strand of chromosome 2 (2q24.3), contains 27 exons, and encodes for the 2005 amino acid voltage-gated sodium channel Na_v_1.2 [41]. The structure of the voltage-gated sodium channel (NaV) highlights its intricate architecture, comprising a pore-forming alpha subunit with four domains and an external IG-fold domain of the beta subunit. The key features include the selectivity filter (DEKA box), the tetrodotoxin (TTX) binding site, and the allosteric coupling enabled by the domain-swapped architecture, which facilitates NaV gating and modulation by blockers such as GX-936 at the voltage-sensor domain (Figure 2) [45]. Na_v_1.2 functions to initiate and propagate action potentials, and de novo truncation or loss of function missense mutations have been recognized as contributing to depression in channel function [46]. This depression has consequently been associated with increased development of ASD and intellectual disability (ID) [41]. Na_v_1.2 is also broadly located throughout the central nervous system but not the peripheral nervous system [47]. These findings explain, in part, why children with this genotypic variant tend to present with delayed motor skills, unsteady/ataxic gait, clumsiness, repetitive actions, and hypotonia [34,46,48]. Studies have established the therapeutic potential of *SCN2A* as a pharmacological target in mitigating the severity of diseases associated with dysfunction of the Na_v_1.2 channel, such as epilepsy, seizures, and ASD/ID [41,49,50]. Brain MRIs from patients with *SCN2A* variants consistently demonstrated structural abnormalities, including agenesis of the corpus callosum, delayed white matter myelination, frontotemporal lobe dysplasia, and enlarged lateral ventricles (Figure 3) [51]. These findings provide critical insights into the neurological impact of *SCN2A* mutations, enhancing our understanding of their role in brain development and predisposition to ASD.

The objective of this manuscript is to systematically review and analyze the genotypic and phenotypic relationships between *SCN2A* gene mutations and ASD, with a focus on elucidating the diverse spectrum of clinical presentations. This systematic review aims to identify specific *SCN2A* variants, including novel mutations, and correlate them with neurological and developmental phenotypes such as seizures, intellectual disability, and motor impairments. Furthermore, the manuscript seeks to explore the potential implications of gender differences and mosaicism in shaping phenotypic variability. Ultimately, this work aims to enhance the understanding of the role of *SCN2A* in ASD, supporting advancements in genetic counseling, personalized medicine, and the development of targeted therapeutic strategies.

## 2. Methods/Materials

### 2.1. Search Strategy

This systematic review was conducted following the guidelines outlined in the Preferred Reporting Items for Systematic Reviews and Meta-Analyses (PRISMA) statement, supplemented by the Cochrane Collaboration Handbook. The review protocol was developed a priori and registered in the international database to register systematic reviews (INPLASY) (registration number: INPLASY202520026). The literature search covered studies published between 2003 and 2024, focusing on the association between *SCN2A* mutations and ASD. The search was conducted using electronic databases, including PubMed, Embase, Web of Science, Scopus, and ScienceDirect.

A comprehensive literature search was conducted for this systematic review on *SCN2A* variants and their correlation with ASD using a combination of Boolean operators, Medical Subject Headings (MeSH), and database-specific search strategies. The primary search terms included “Autism Spectrum Disorder” [Mesh], “*SCN2A*”, and “Humans” [Mesh], ensuring a broad yet relevant capture of studies examining the role of *SCN2A* in neurodevelopmental disorders. To refine the search, additional terms such as “*SCN2A* mutations”, “Nav1.2 sodium channel”, “genotype-phenotype correlation”, and “*SCN2A* variant spectrum” were incorporated. Variations in clinical presentations were also considered by including terms such as “neurodevelopmental delay”, “cognitive impairment”, “intellectual disability”, “seizures”, and “epilepsy”. Furthermore, since the study explored mosaicism and gender differences in phenotypic expression, relevant terms such as “*SCN2A* mosaicism”, “sex differences in ASD”, and “gender-specific genetic impact” were included.

### 2.2. Study Selection

The inclusion criteria for this review were studies focusing on *SCN2A* gene variations and their clinical implications in ASD. Eligible studies included those that analyzed genetic associations, case–control studies, and case reports/series involving human subjects of any age who have been diagnosed with ASD using gold-standard diagnostic tools. Only peer-reviewed studies published in English were included. Studies that focused on other conditions, those without clinical imaging or genetic data, animal or in vitro studies, non-English articles, and those lacking sufficient methodological rigor were excluded.

### 2.3. Data Extraction

Data extraction was conducted independently by a team of reviewers (ND, JC, DE, MZ, DC, JM, and AM) who applied predetermined inclusion and exclusion criteria. In cases of disagreement, another reviewer or senior author was consulted to resolve discrepancies. Extracted data included patient demographics (age, sex), the presence or absence of an ASD diagnosis, genetic variations, and association statistics (such as *p*-values) assessing the correlation between *SCN2A* mutations and ASD. Data were recorded in an Excel sheet.

### 2.4. Risk of Bias and Quality Assessment

The risk of bias was assessed using the Joanna Briggs Institute (JBI) critical appraisal checklist, tailored to the study type [52,53,54,55]. The GRADE criteria and the Risk of Bias Assessment tool for Non-Randomized Studies (RoBANS) were also applied to formally assess the quality of evidence and potential bias in the included studies. Three researchers (ND, JC, and DE) critically reviewed the included studies for scientific quality and control of confounders, with discrepancies resolved by discussion and consensus or discussion with the senior author.

### 2.5. Data Synthesis

Data from studies that met the inclusion criteria were synthesized and presented in both tabular and narrative formats. The characteristics, methods, outcomes, and study quality were described. Data were reported as medians, percentages, ranges, and means with standard deviations, where applicable. Subgroup analyses were performed based on sex (male vs. female) and age groups to explore any differential effects of *SCN2A* mutations in these populations.

## 3. Results

**Figure 4:**
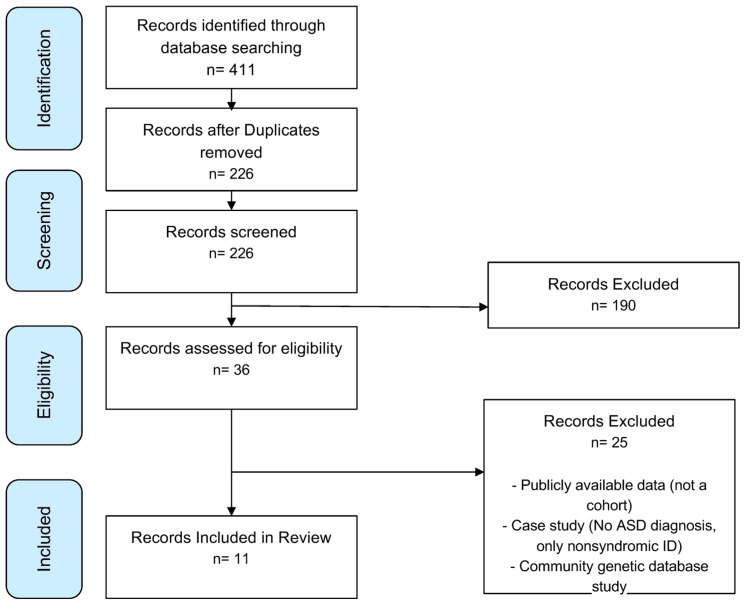
**PRISMA flow diagram for study selection:** A PRISMA (Preferred Reporting Items for Systematic Reviews and Meta-Analyses) flow diagram illustrating the systematic review process, including the identification, screening, eligibility, and inclusion of studies. The diagram details the number of records retrieved, duplicates removed, records screened, full-text articles assessed, excluded studies, and final studies included in the review.

**Figure 5:**
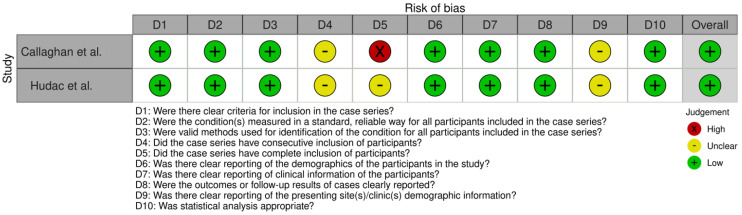
**Risk of bias analysis for case series studies:** Assessment of risk of bias in the included case series studies using the Joanna Briggs Institute (JBI) checklist. The analysis provides a detailed evaluation of methodological rigor, with color coding to denote the level of risk: green for low risk, yellow for unclear risk, and red for high risk, highlighting areas of strength and potential limitations across key domains [57,58].

**Figure 6:**
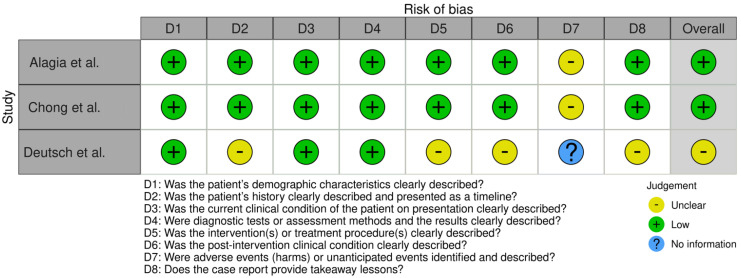
**Risk of bias analysis for case report studies:** Assessment of risk of bias in the included case report studies assessed using the Joanna Briggs Institute (JBI) checklist. The evaluation identifies key strengths and limitations across critical domains, with a color-coded system indicating risk levels: green for low risk and yellow for moderate or unclear risk, and blue for no information [59,60,61].

**Figure 7:**
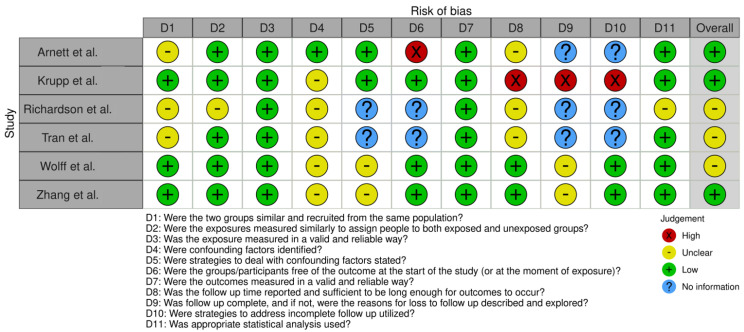
**Risk of bias analysis for cohort studies:** Assessment of risk of bias in the included cohort studies assessed using the Joanna Briggs Institute (JBI) checklist. The analysis provides a detailed evaluation of methodological rigor, with color coding to denote the level of risk: green for low risk, yellow for unclear risk, red for high risk, and blue for no information, highlighting areas of strength and potential limitations across key domains [49,56,62,63,64,65].

This systematic review followed the PRISMA guidelines to ensure a rigorous selection process for identifying relevant studies. Searches were conducted in the PubMed, Embase, Web of Science, and Scopus databases, resulting in 411 initial records (Figure 4). After removing duplicates, 226 unique records were screened based on predefined inclusion and exclusion criteria, such as focusing on human participants, examining gene associations with ASD, and being published in English between 2017 and 2023. Of these, 190 studies were excluded during the screening phase for not meeting the criteria. A further 36 studies underwent eligibility assessment, leading to the exclusion of 25 studies due to reliance on publicly available data, duplication of published findings, inappropriate case studies (such as lacking ASD diagnoses), or irrelevant nonsyndromic intellectual disability cases. Ultimately, 11 studies met the inclusion criteria, offering valuable insights into genetic factors associated with ASD (Table 1). The articles encompassed a total of 695 patients diagnosed with ASD. Among these patients, 75 (10.8%) were identified as having *SCN2A* mutations, highlighting the prevalence of this genetic variation in the ASD population. The studies varied in size, with patient cohorts ranging from single case reports to large groups, such as the 354 patients analyzed in the study by Zhang et al. (2023) [56]. The risk of bias (RoB) analysis for cases series, case reports, and cohort studies is outlined in Figure 5, Figure 6, and Figure 7 respectively. The studies included in this systematic review were determined to be of high methodological quality, with a low risk of bias, ensuring their appropriateness for inclusion in the analysis.

**Table 1:** Overview of included studies. Summary of studies reporting SCN2A variants in ASD cohorts, including sample size, diagnostic tools, and demographic details.

Reference	Observations	N (Total)	N (ASD)	N (ASD + SCN2a)	ASD Diagnosis Tool	Ethnicity	Race	M	F	Age (Range)	Consanguinity
Alagia et al. [59]	Includes dystonic movements	1	1	1	NR	African	Black		1	14	NR
Arnett et al. [63]	Subset includes SCN2A cases	65	45	10	ADI-R	NR	NR	4	6	5–21	NR
Callaghan et al. [58]	Includes detailed sequencing info	119	119	2	DSM-IV-TR, ADI-R, ADOS-G	NR	NR	2		13–23	NR
Chong et al. [60]	SCN2A and SCN3A deletions	1	1	1	M-CHAT-R	NR	NR	1		1.75	NR
Deutsch et al. [61]	Case report, detailed phenotype	1	1	1	Clinical diagnosis	NR	NR	1		29	NR
Hudac et al. [57]	Explores sensory phenotypes	39	24	24	NR	33 White, 5 Hispanic	Mixed (Asian, African)	19	20	3–22	NR
Krupp et al. [64]	Focuses on mosaic mutations	22	14	7	DSM-V	White, African American, Asian	NR	14	8	3–15	NR
Richardson et al. [65]	Detailed phenotypic spectrum	22	3	3	Not specified	White, Asian, Middle Eastern	NR	10	12	2–52	NR
Tran et al. [62]	Vietnamese cohort	100	100	1	DSM-V	Vietnamese	NR	0	1	3.5	NR
Wolff et al. [49]	Large cohort with heterogeneity	71	23	10	NR	NR	NR	NR	NR	NR	NR
Zhang et al. [56]	Pipeline analysis for SCN2A mutations	354	354	3	DSM-V	NR	NR	279	75	NR	NR

NR: Not reported.

### 3.1. Patient Population and Diagnosis

The patient demographics in studies involving *SCN2A* mutations ASD demonstrated a diverse range of age groups, ethnicities, and sample sizes. Patient ages ranged from infancy (1.75 years) to adulthood (52 years), with both male and female participants represented, although gender-specific data were often incomplete. Ethnic representation was variable, with cohorts including African, Asian, Middle Eastern, Hispanic, and Vietnamese populations, alongside White individuals. Some studies, such as that by Hudac et al. [57], specifically observed ethnic diversity, including mixed racial groups, whereas others, such as that by Tran et al. [62], focused on more homogenous populations such as Vietnamese children. Gender breakdowns, where available, revealed disparities in male-to-female ratios; for instance, Zhang et al. [56] reports 279 males and 75 females in their cohort. However, consanguinity, a potential factor influencing genetic mutations, was not explicitly reported in most studies. This demographic variability highlights the heterogeneity of patient populations studied in the context of *SCN2A*-related ASD.

### 3.2. Phenotypic Data

Seizures and epilepsy are among the most common features of *SCN2A*-associated cases. In the study by Alagia et al. [59], seizures were present in the single reported patient, alongside late-onset generalized epilepsy. Similarly, Richardson et al. [65] documented seizures in 16 out of 22 patients (72.7%), with epilepsy noted in multiple individuals, further highlighting the strong association between *SCN2A* mutations and seizure disorders. However, in some studies, such as that by Tran et al. [62], seizures were notably absent in the cohort, illustrating phenotypic variability across cases.

Psychiatric manifestations, including anxiety, hyperactivity, and behavioral disturbances, were documented in several cases. Richardson et al. [65] described a subset of patients exhibiting behavioral issues such as aggression and obsessive behaviors. Meanwhile, Hudac et al. [57] reported repetitive actions such as stereotypic rocking and echolalia in their cohort. These findings suggest that psychiatric and behavioral features may represent an important subset of *SCN2A*-related phenotypes.

Physical abnormalities were also a recurring theme in the analyzed data. For example, Chong et al. [60] described facial dysmorphism in their single patient, while Hudac et al. [57] identified microcephaly. Additional abnormalities, such as limb anomalies, were also noted in the cohort analyzed by Richardson et al. [65], emphasizing the structural and developmental impact of *SCN2A* mutations.

Developmental delays (DDs) and communication impairments were consistently documented across studies. In the report by Wolff et al. [49], moderate-to-severe developmental delays were observed in all cases, with speech delays highlighted in a significant proportion. Similarly, Hudac et al. [57] identified communication impairments in a majority of their cohort, including 12 patients with significant verbal deficits and 27 patients categorized as nonverbal. These findings align with the broader understanding of *SCN2A* mutations as a significant contributor to neurodevelopmental impairments.

The phenotypic data (Table 2) reveal consistent trends in *SCN2A*-associated conditions, including a high prevalence of seizures, epilepsy, psychiatric and behavioral features, physical abnormalities, and developmental delays. The variability observed across cases—both in severity and specific manifestations—likely reflects the diverse genetic and environmental factors influencing clinical outcomes. These findings highlight the importance of *SCN2A* as a critical gene implicated in neurodevelopmental disorders and provide a foundation for further research into targeted therapeutic strategies.

### 3.3. Genotypic Data

The analysis of genetic mutations associated with *SCN2A*-related disorders reveals a diverse spectrum of variant types, each contributing differently to clinical presentation (Table 3 and Table 4).

Among the studies reviewed, missense mutations were the most frequently observed, with a total of 58 instances. Hudac et al. [57] and Wolff et al. [49] reported 18 and 13 missense variants, respectively. These mutations were commonly associated with neurodevelopmental phenotypes, including developmental delay, ASD, and sensory processing abnormalities. Representative examples include p.Arg102Gln (Callaghan et al. [58]), p.A704K (Deutsch et al. [61]), and p.Asp12Asn and p.Gly822Ser (Arnett et al. [63]).

Frameshift and nonsense mutations were each identified in 14 cases. Frameshift variants, described by Arnett et al. [63] and Krupp et al. [64], frequently resulted in premature termination of the SCN2A protein and were associated with intellectual disability and global developmental delay. Similarly, nonsense mutations, including five reported by Richardson et al. [65], were linked to a range of cognitive and behavioral impairments. Splicing mutations were observed in nine cases. Alagia et al. [59] described a novel splicing variant, c.2919+4delT, which resulted in abnormal exon inclusion and was predicted to disrupt SCN2A channel function. This mutation was associated with low-functioning ASD and late-onset epilepsy.

Structural deletions were reported in five individuals, most notably a 1.1 megabase deletion encompassing both *SCN2A* and *SCN3A*, as documented by Chong et al. [60]. This large-scale genomic alteration was associated with severe clinical phenotypes, including West syndrome and ASD. In addition, 10 single nucleotide variants were identified, primarily by Arnett et al. [63], who reported their association with specific ASD subtypes. Zhang et al. [56] reported a deletion variant, c.4550_4551del, in a large cohort of Chinese individuals with ASD, highlighting the importance of population-specific genetic findings.

In terms of inheritance patterns, de novo mutations were the most common, with 66 instances reported across the studies. These findings emphasize the largely sporadic nature of *SCN2A*-related conditions. Wolff et al. [49] described several de novo mutations, including Q1811E and M1548V. Mosaicism was observed in three cases, including presumed gonadal mosaicism in maternal half siblings, as described by Richardson et al. [65]. Most variants were heterozygous, with 49 instances reported, reflecting the clinical relevance of single-allele disruptions in *SCN2A*.

These findings illustrate the complexity of the genetic landscape in *SCN2A*-related disorders. The wide range of variant types, including missense, nonsense, frameshift, splicing, and structural mutations, contributes to the broad phenotypic variability observed in affected individuals.

## 4. Discussion

### Analysis of Genotypic and Phenotypic Trends

The data analyzed in this study provide significant insights into the genotypic and phenotypic spectrum associated with *SCN2A* mutations in patients with ASD. Among the 695 patients across 11 studies, 75 (10.8%) were identified as having *SCN2A* mutations. Of these, 58 cases involved missense mutations, making it the most prevalent mutation type. A missense mutation occurs when single-nucleotide changes result in the substitution of one amino acid for another in the protein sequence, potentially altering its structure and function. This trend aligns with the known impact of missense mutations on the function of the Nav1.2 sodium channel, which plays a critical role in neuronal excitability within the central nervous system. Nav1.2 channels are responsible for initiating and propagating action potentials in neurons, facilitating rapid communication between nerve cells [66]. When these channels are underutilized or their function is depressed, it can lead to reduced neuronal activity, which is associated with developmental delays, intellectual disabilities, and conditions like ASD [50]. Conversely, overactivation of these channels can result in excessive neuronal firing, potentially leading to seizures and other hyperexcitable states, further contributing to the neurological symptoms observed in individuals with *SCN2A* mutations [66,67,68,69].

Frameshift mutations, which involve insertions or deletions of nucleotides that disrupt the reading frame of the gene, were the next most common, found in 14 patients, followed by 14 cases of nonsense mutations, which introduce a premature stop codon, leading to truncated, nonfunctional proteins. Both typically lead to severe phenotypes, including profound intellectual disability and intractable epilepsy due to the loss of function in the *SCN2A* protein. Splicing mutations, which affect the normal removal of introns and assembly of exons during RNA processing, were documented in nine patients, highlighting the complexity of *SCN2A*-related disorders and the potential for these mutations to result in abnormal RNA processing.

Phenotypically, individuals with *SCN2A* mutations frequently exhibited a combination of motor impairments and behavioral symptoms characteristic of ASD [41]. Specifically, 19 patients reported difficulties with walking, including unsteady or ataxic gait, and 29 patients exhibited movement disorders like hypotonia and dystonia. Behavioral symptoms included repetitive actions and delayed speech development, with 22 patients experiencing speech delays and 17 reported as nonverbal. Additionally, seizures and epilepsy were prevalent, affecting 79 and 37 patients, respectively, further underscoring the neurological impact of *SCN2A* mutations. Previous studies have extensively documented the association between *SCN2A* mutations and epilepsy, particularly in early-onset cases. For instance, Wolff et al. and Ben-Shalom et al. found that mutations in *SCN2A* can lead to both gain-of-function and loss-of-function effects in the Nav1.2 sodium channel, contributing to hyperexcitability in neurons and resulting in seizure activity [49,50]. These studies emphasize that *SCN2A*-related epilepsy often presents within the first few months of life and can be severe, sometimes resistant to standard antiepileptic treatments [41]. The observed phenotypic variability, even among patients with similar genotypic alterations, suggests the influence of other genetic, epigenetic, or environmental factors on clinical presentation. Moreover, the association of *SCN2A* mutations with early-onset epilepsy and intellectual disability, observed in 79 and 65 patients, respectively, corroborates findings from previous studies such as those by Ben-Shalom et al. and Christensen et al. [44,50]. The phenotypic variability documented in our data, where some patients exhibit mild forms of ASD while others present with severe neurodevelopmental disorders, mirrors the heterogeneity noted in earlier research [39,70]. This variability is particularly evident in the context of gender, where the impact of *SCN2A* mutations appears to differ between males and females, a pattern that has been reported in other genetic studies on ASD [3,35,66].

An important aspect of the data concerns the gender distribution of patients with *SCN2A* mutations. Among the 201 patients for whom gender data were available, there were 114 males and 87 females, indicating a slight male predominance. While this cohort demonstrated a slight male predominance, the distribution of males and females in the cohort was skewed away from the typical 4:1 ratio of males to females [71]. This may indicate *SCN2A* affecting a female population more than other known ASD genetic mutations [72]. The higher prevalence of *SCN2A* mutations in males may reflect this broader epidemiological pattern, suggesting that males may be more susceptible to the neurodevelopmental impacts of *SCN2A* mutations.

The reason for this male predominance in ASD and related genetic conditions like those involving *SCN2A* is not fully understood, but it is thought to involve a combination of genetic, hormonal, and possibly environmental factors [73]. Some researchers have proposed that females might have a higher threshold for developing ASD, meaning that they require a greater mutational burden or additional genetic and environmental risk factors to exhibit ASD symptoms compared to males [43,71,74]. This could explain why the females in this cohort, although fewer in number, might present with more severe phenotypes, though this hypothesis requires further investigation. There is also a thought that females may display a differing array of symptoms, which can be more subtle than in their male counterparts [71]. The current diagnostic understanding of ASD is based largely on studies and observations in males, possibly allowing affected females to be missed by current diagnostic criteria [32,33].

Although this review documents gender distribution and reports genotype–phenotype associations, a detailed statistical analysis of the most frequent SCN2A variants among individuals with comorbid ASD and epilepsy, as well as formal testing of gender-specific trends, was not performed. These analyses are important and could yield meaningful insights, but they were beyond the scope of this study. Future investigations using harmonized datasets and larger cohorts may be better positioned to explore these relationships more robustly.

The trends observed in this study align with findings from previous research on *SCN2A*-related disorders. Prior studies have consistently identified *SCN2A* as a significant contributor to ASD, with a strong correlation between *SCN2A* mutations and severe neurological phenotypes [5,39]. For instance, the predominance of missense mutations (58 cases) aligns with reports from Sanders et al. and Wolff et al., which highlight the role of these mutations in disrupting Nav1.2 channel function, leading to neurological symptoms associated with ASD and related conditions [41,49].

While the overall trends in this study align with the existing literature, our analysis contributes to the growing body of knowledge by providing a more detailed examination of specific genotypic and phenotypic correlations within a larger cohort of patients. The documentation of specific mutations, such as the novel splicing mutation c.2919+4delT, and their corresponding clinical manifestations adds valuable information to the understanding of *SCN2A*-related disorders.

One of the novel aspects of this study is the detailed examination of specific *SCN2A* mutations and their precise phenotypic outcomes, including a focus on gender differences. While previous studies have identified the general association between *SCN2A* and ASD, this study goes further by cataloging exact mutations and linking them to specific clinical presentations, such as low-functioning autism and dystonic movement disorder associated with the c.2919+4delT splicing mutation. This level of detail enhances our understanding of how different mutations within the *SCN2A* gene contribute to the spectrum of ASD phenotypes and how these effects might differ between males and females.

The identification of mosaicism in three cases also adds a new dimension to the understanding of *SCN2A*-related disorders. Mosaic mutations, where different cells within the same individual have different genetic makeups, may explain some of the phenotypic variability observed in patients [45]. This finding suggests that mosaicism could contribute to the heterogeneity of clinical presentations in *SCN2A*-related ASD, an area that has not been extensively explored in previous research [75,76]. Furthermore, the focus of the study on the implications of these findings for genetic counseling and targeted therapy is particularly novel. By establishing a clearer genotype–phenotype correlation, including gender-specific trends, this research supports the potential for personalized treatment strategies that target specific mutations within the *SCN2A* gene. This approach could lead to more effective interventions for individuals with *SCN2A*-related ASD, particularly given that 66 of the mutations in this study were de novo, indicating spontaneous genetic alterations that could be specifically targeted in therapeutic contexts.

In conclusion, while this study’s findings largely align with the existing literature, it makes several novel contributions to the field by providing a detailed genotype–phenotype analysis, identifying gender-specific trends and the role of mosaicism, and highlighting the potential for personalized therapeutic approaches. These insights not only enhance our understanding of *SCN2A*-related disorders but also pave the way for future studies aimed at improving outcomes for individuals with ASD linked to *SCN2A* mutations.

## 5. Limitations

This systematic review provides critical insights into the relationship between *SCN2A* mutations and ASD; however, several limitations must be acknowledged. First, the studies included in this review exhibit variability in their methodologies, particularly in the diagnostic criteria for ASD and the genetic screening approaches used, which may have introduced heterogeneity into the data. Second, the reliance on retrospective data and case reports limits the ability to establish causality between specific *SCN2A* mutations and phenotypic outcomes. Third, the relatively small sample size for certain subgroups, such as patients with mosaic mutations or novel variants, constrains the generalizability of these findings to the broader ASD population.

Lastly, while this review highlights the potential for targeted therapeutic strategies, the absence of prospective, large-scale studies to validate these approaches underscores the need for future research. Comprehensive investigations that include diverse populations, standardized methodologies, and functional analyses of *SCN2A* mutations are essential to overcome these limitations and advance the understanding of *SCN2A*-related ASD. Despite these constraints, this systematic review provides a foundation for further exploration of the role of *SCN2A* in ASD pathophysiology and therapeutic development.

## 6. Conclusions and Future Directions

This systematic review highlights the critical role of *SCN2A* mutations in ASD, shedding light on the intricate genotypic and phenotypic relationships that define its clinical manifestations. By analyzing data from 695 patients across 11 studies, the findings highlight the prevalence of *SCN2A* mutations in ASD and their significant contribution to diverse neurological symptoms, including seizures, intellectual disability, motor impairments, and behavioral deficits. The identification of specific mutation types, such as missense mutations, frameshifts, and splicing abnormalities, and their association with distinct phenotypes provides valuable insights into the molecular underpinnings of ASD. Furthermore, the exploration of gender differences and mosaicism reveals additional layers of complexity in the clinical presentation, underscoring the need for personalized approaches in diagnosis and treatment.

The findings presented here hold substantial clinical significance. By detailing genotype–phenotype correlations, this work provides a foundation for more precise genetic counseling and risk assessment for families affected by *SCN2A*-related disorders. Additionally, the identification of specific mutations, including novel variants, points to opportunities for the development of targeted therapies, particularly for severe manifestations such as early-onset epilepsy and profound intellectual disability. These insights could pave the way for precision medicine approaches that align therapeutic interventions with individual genetic profiles, improving outcomes for affected individuals.

Future research directions include prospective, large-scale studies to validate genotype–phenotype correlations and further explore the impact of gender differences and mosaicism in *SCN2A*-related ASD. Functional studies investigating the biological effects of specific *SCN2A* mutations will be crucial in identifying druggable targets and understanding the molecular mechanisms driving phenotypic variability. Additionally, integrating emerging technologies such as CRISPR-based gene editing and patient-derived neuronal models will allow for preclinical testing of novel therapeutic strategies tailored to *SCN2A*-related disorders [11].

Expanding the diversity of study populations will also be essential to ensure the generalizability of findings across ethnic and demographic groups. The interdisciplinary collaboration among geneticists, neurologists, and behavioral scientists will be pivotal in translating these discoveries into clinical practice, ultimately improving the quality of life for individuals with *SCN2A*-related ASD and their families.

## Figures and Tables

**Table 2:** Phenotypic data. This table summarizes the clinical features of *SCN2A*-related cases, highlighting the prevalence of seizures, epilepsy, and other neurological manifestations.

Reference	Seizures	Epilepsy	Psychiatric	Repetitive Actions	Verbal	Nonverbal	Speech Delay	Developmental Delay (DD)	ID	Physical Abnormalities	Movement Disorders	Dysphagia	Walking	Crawling	Unsteady/Ataxic Gait
Alagia et al. [59]	Yes	Yes	Anxiety	Pelvis rocking	1	1	Severe	Yes	NR	Facial dysmorphism	Dystonia	NR	NR	NR	Yes
Arnett et al. [63]	No	No	NR	Echolalia	5	2	Moderate	No	NR	None	None	NR	Yes	Yes	NR
Callaghan et al. [58]	Yes	No	Hyperactivity	None	3	NR	Mild	Yes	NR	Microcephaly	Hypotonia	NR	No	NR	Yes
Chong et al. [60]	Yes	Yes	NR	Stereotypic rocking	1	NR	Severe	Yes	NR	Dysmorphic features	None	NR	Yes	Yes	Yes
Deutsch et al. [61]	No	No	NR	NR	NR	NR	NR	NR	NR	NR	NR	NR	NR	NR	NR
Hudac et al. [57]	Yes	NR	NR	Echolalia	2	5	Severe	Yes	NR	Microcephaly	Hypotonia	NR	Yes	NR	Yes
Krupp et al. [64]	No	NR	Behavioral issues	NR	NR	NR	NR	Yes	NR	None	None	NR	No	No	NR
Richardson et al. [65]	Yes	NR	Aggression	Stereotypy	5	6	Severe	Yes	NR	Dysmorphic features	NR	NR	Yes	NR	Yes
Tran et al. [62]	No	No	Hyperactivity	None	NR	NR	Mild	No	NR	None	None	NR	NR	NR	NR
Wolff et al. [49]	Yes	Yes	NR	NR	NR	NR	Severe	Yes	NR	Microcephaly	Ataxia	NR	NR	NR	Yes
Zhang et al. [56]	No	None	None	None	NR	NR	None	No	NR	None	None	NR	NR	NR	NR

NR: Not reported.

**Table 3:** Overview of genetic mutations in SCN2A-related disorders. Summary of inheritance patterns, zygosity, and predicted functional effects (GoF = gain of function, LoF = loss of function).

Reference	Mutation Type	Homozygous/ Heterozygous	Maternal Inheritance Paternal Inheritance	Unknown Origin	Mosaic	De Novo	Exon	Intron	GoF	LoF
Alagia et al. [59]	1 (Splicing)	0	0	0	0	0	0	1	0	0
Arnett et al. [63]	2 (Frameshift), 6 (Missense), 1 (Nonsense), 1 (Splicing), 10 (SNV)	10 Heterozygous	0	3	0	7	6	0	0	0
Callaghan et al. [58]	1 (Missense), 1 (Splicing), 2 (SNV)	0	0	0	0	2	0	0	0	1
Chong et al. [60]	1 (Deletion)	0	0	0	0	1	0	0	0	0
Deutsch et al. [61]	1 (Missense), 1 (SNV)	0	0	0	0	1	0	0	0	0
Hudac et al. [57]	3 (Frameshift), 18 (Missense), 5 (Nonsense) 1 (Splicing), 1 (Duplication), 1 (Stop/Gain)	0	2 (Paternal)	7	0	26	0	0	0	0
Krupp et al. [64]	1 (Deletion), 2 (Frameshift), 5 (Missense), 2 (Nonsense), 1 (Splicing), 1 (Stop/Gain Included in Nonsense), 9 (SNV)	9 Heterozygous	1 (Maternal)	0	2	7	11	0	0	Implicated in frameshift and nonsense mutations
Richardson et al. [65]	2 (Deletion), 2 (Frameshift), 14 (Missense), 5 (Nonsense), 3 (Splicing)	0	1 (Maternal) 1 (Paternal)	2	2	18	0	0	0	6
Tran et al. [62]	1 (Frameshift)	0	0	0	0	1	1	0	0	1
Wolff et al. [49]	5 (Frameshift), 13 (Missense), 2 (Nonsense), 2 (Splicing)	NR	0	0	0	22	20	2	NR	NR
Zhang et al. [56]	1 (Deletion), 1 (Frameshift), 1 (Nonsense), 1 (Splicing)	NR	0	0	0	3	2	1	NR	NR

NR: Not reported.

**Table 4:** Specific genetic mutations in SCN2A studies. This table summarizes reported SCN2A variants across multiple studies, including the specific genetic changes, associated protein alterations, and relevant notes such as inheritance patterns or variant types. Variants include missense, nonsense, splice-site, frameshift, and large deletions.

Reference	Variant(s)	Notes
Alagia et al. [59]	c.2919+4delT	NM_001040142
Arnett et al. [63]	c.34G>A, c.2464G>A, c.2645G>A, c.2877C>A, c.2932T>C, c.4264A>G, c.4896_4897insT, c.4996C>T, c.425delA, c.605+1G>T	Multiple variants
Callaghan et al. [58]	p.Arg102Gln, splice acceptor variant	Missense and splicing
Chong et al. [60]	1.1 Mb deletion including SCN2A and SCN3A	Large deletion
Deutsch et al. [61]	p.A704K	Alanine to lysine
Hudac et al. [57]	c.34G>A, c.1289A>C, c.5339G>T, c.2932T>C, c.5318C>T, c.5272A>C, c.2877C>A, c.605+1G>T, c.3849+2T>C, c.4801G>T, c.2635G>A, c.4938_4939insGAT, c.4591C>T, c.823C>T, c.252C>A, c.1712G>A, c.632G>A, c.2464G>A	Multiple variants
Krupp et al. [64]	c.3370A>T, c.272A>G, c.1094C>T, c.3922C>T, c.1819C>T, c.5536C>T, c.3435_3436delCG, c.469delA, c.1384+1G>T	Multiple types
Richardson et al. [65]	c.2932T>C, c.2674G>A, c.5192G>A, c.4644G>C, c.640T>G, c.2774T>C, c.5318C>T, c.1184G>A, c.4543C>T, c.515T>G, c.4780T>A, c.4886G>A, c.5638G>A	Multiple variants
Tran et al. [62]	p.Leu78fs	De novo
Wolff et al. [49]	Q1811E, M1548V, E430A, E999K, R1319Q (x2), R1882P, A1652P, H930Q, P1622S, c.605+1G4T, V1528Cfs*7, C1170Vfs*15, R1235*, A1773V, K1933M, N503Kfs*19, K1387Sfs*4, R1435*, T1711Lfs*8, G1744E, c.386+2T>C	All de novo
Zhang et al. [56]	c.4550_4551del, c.605+1G>A, c.1570C>T	Subjects 3F, 3M, 4M
